# Relationship between social support and parenting sense of competence in puerperal women: Multiple mediators of resilience and postpartum depression

**DOI:** 10.3389/fpsyt.2022.986797

**Published:** 2022-10-14

**Authors:** Xingchen Shang, Lin Li, Changmin Niu, Yuexia Liao, Shuang Gao

**Affiliations:** ^1^School of Nursing, Yangzhou University, Yangzhou, China; ^2^School of Nursing, The University of Hong Kong, Hong Kong SAR, China; ^3^Department of Nursing, Affiliated Hospital of Yangzhou University, Yangzhou, China; ^4^School of Health Management, Guangzhou Medical University, Guangzhou, China

**Keywords:** social support, maternal role competence, resilience, postpartum depression, mediation effect

## Abstract

**Background:**

Maternal role competence is an important marker of achievement in the role of the mother, but parenting sense of competence (PSOC) among puerperal women is not high. Psychosocial factors, particularly social support, postnatal depression and resilience, have been identified as significant predictors of maternal role competence. However, information is limited regarding the mechanisms through which these psychosocial factors affect maternal role competence.

**Objective:**

To evaluate the multiple mediators of resilience and postpartum depression (PPD) in the relationship between social support and PSOC in puerperal women.

**Methods:**

A cross-sectional study was performed in a tertiary general hospital in Yangzhou, China. A total of 234 puerperal women at 6–8 weeks after birth completed the socio-demographic questionnaires, Social Support Rating Scale, Connor–Davidson Resilience Scale, Edinburgh Postnatal Depression Scale, and PSOC Scale.

**Results:**

Resilience and PPD mediated the relationship between social support and PSOC. The mediation effect of resilience and PPD and the total mediation effect were significant, individually accounting for 22.96, 21.70, and 44.65%, respectively, of the total effect. Moreover, pairwise contrast between the two indirect effects was not significant. The difference between the two pathways suggests that resilience and PPD play different roles in the relationship between social support and PSOC.

**Conclusions:**

This study showed that social support may exert its effects on PSOC in puerperal women with multiple mediators of resilience and PPD. This therefore highlights potential intervention targets to improve PSOC.

## Introduction

Puerperium is an important period because mothers need to recover and transform their self-role to the role of mothers. The term, ‘maternal role competence' refers to the belief in one's ability to effectively perform nurturing behaviors, while perceived maternal role satisfaction is related to the perceptions of pleasure and gratification derived from the maternal role (1). Achieving maternal role competence is considered one of the most critical components of maternal adaptation, affecting parenting behaviors and the psychosocial development of children (2, 3). However, mothers are often found to express feelings of inadequacy (2), and studies have shown that a woman's parenting sense of competence (PSOC) in puerperium is only moderate (4). Maternal role competence during the transition to motherhood can have a tremendous impact on the quality of parenting behaviors. Thus, it is important that the factors influencing parental role competence are understood.

Psychosocial factors, support, postnatal depression, and resilience have been identified as significant predictors of maternal role competence (5, 6). However, information is limited on the mechanisms through which these psychosocial factors affect maternal role competence. Social support has been reported as a significant predictor of parenting competence, and previous research has demonstrated that parents with a higher level of social support have a higher level of parental role competence (2, 7, 8). Social support refers to interpersonal transactions that provide individuals with esteem, stress-related aid, and emotional assistance (9). It is one of the most important factors impacting positively on women's sense of well-being in the postpartum period. A randomized controlled trial from China showed that enhancement in social support could have a positive effect on the maternal role competence of new mothers (10). This result is in accordance with Bandura's self-efficacy theory. Bandura (11) proposed that parents who perceive higher levels of support from their family (more verbal encouragement and parenting skills assistance) have improved parental role competence.

Postpartum depression (PPD) is defined as a major depressive episode, which occurs during pregnancy or in the 4 weeks following delivery (12). However, in clinical practice and research, PPD is variably defined as occurring from 4 weeks to 12 months after childbirth (12). PPD, which affects 10–20% of women globally and is more common in low-income regions (13, 14), is the main factor influencing women's PSOC in puerperium (4). Previous evidence has suggested that social support is a significant predictor of PPD (15). Emotional support and practical assistance in childcare from family members, friends, and professionals may help women adapt to stress and emotional challenges during the early weeks of motherhood, thereby minimizing the risk of PPD. A comparative study of the predictors of postnatal depression in Taiwan and mainland China found that social support is a significant predictor among 197 women living in mainland China (16).

Resilience is a subjective measure of the capacity to adapt to adversities in life, and encompasses concepts such as inner strength, competence, and flexibility (17). Resilience has been shown to have a protective effect on obstetric and infant outcomes (18). Additionally, enhancing resilience may be an important component in improving PSOC (6). However, evidence of resilience in puerperal women in mainland China is relatively limited. Thus, the relationship among social support, resilience, and PSOC remains unclear.

In mainland China, the number of new births has increased rapidly with the implementation of the ‘universal two-child' policy (19). The increasing total number of newborns has increased the number of women in the postpartum period, thereby causing new challenges and pressures for maternal and child health services. Accordingly, the mental health of pregnant women has become a focus of research. As women's experience of mothering integrates multiple factors that should be appraised in the cultural context (2), Chinese cultural attitudes to maternal role competence should be understood. Although past studies have suggested that social support and resilience may serve as protective factors, and PPD may serve as a risk factor, limited information is available on the mechanisms through which these factors affect maternal role competence during the transition to early motherhood, particularly among Chinese populations. Moreover, understanding the relationship between social support and maternal role competence is imperative for the development of effective interventions to promote the latter.

**Figure 1:**
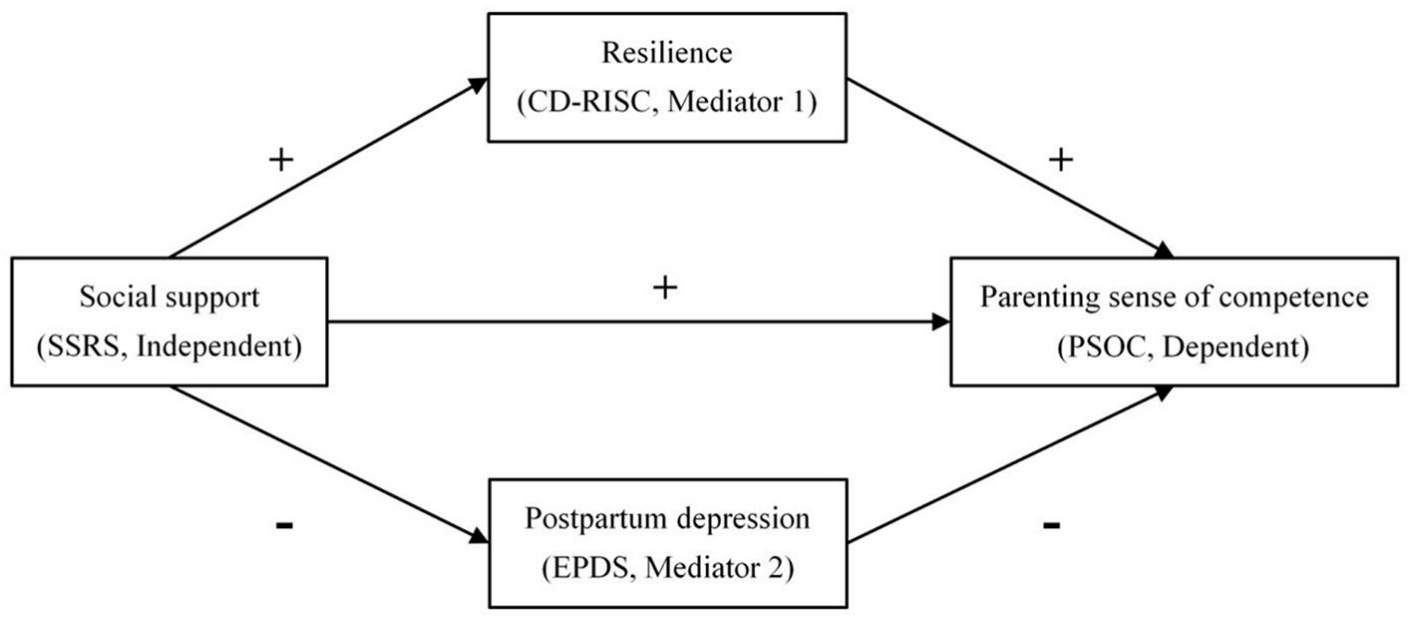
Conceptual framework of the social support, resilience, postpartum depression and parenting sense of competence among puerperal women.

On the basis of a literature review, a conceptual model of social support, resilience, PPD and PSOC among puerperal women was constructed (Figure 1). The following hypotheses were formulated:

**Hypothesis 1**. Social support is positively correlated with PSOC.
**Hypothesis 2**. Resilience is positively correlated with PSOC.
**Hypothesis 3**. Postpartum depression is negatively correlated with PSOC.
**Hypothesis 4**. Resilience plays a mediating role between social support and PSOC.
**Hypothesis 5**. Postpartum depression plays a mediating role between social support and PSOC.

The aims of this study are as follows: (1) explore the relationship among social support, resilience, PPD, and PSOC; (2) examine whether or not resilience can mediate between social support and PSOC and (3) test whether or not PPD can mediate between social support and PSOC.

## Materials and methods

### Recruitment and participants

This cross-sectional study was conducted between May 6 and August 20, 2020. Participants were recruited from the obstetric clinic in a tertiary general hospital in the city of Yangzhou, Jiangsu Province. Inclusion criteria for participants were as follows: puerperal women whose delivered newborns were normal full-term babies; follow-up visit at a postpartum outpatient clinic within 6–8 weeks postpartum; at least 18 years of age; and hometown in mainland China. Exclusion criteria were as follows: pregnancy complications or complications (e.g., diabetes, hypertension, heart disease); postpartum complications; mental or psychological diseases.

All women included in the study voluntarily participated and all signed an informed consent form. For the structural equation modeling (SEM) analysis, the minimum sample size was calculated to be over 200 (20). However, based on a 15% dropout rate, it was determined that a sample size of 230 would be needed.

The participants were informed of the purpose and significance of the study before the investigation. Eligible puerperal women were invited to complete the pen-and-paper survey in a quiet room near the obstetrics clinic. A nurse with a graduate degree was responsible for the data collection. All questionnaires were checked on-site immediately after completion to identify any missing data.

### Measures

#### Social support

Social support was assessed using the Social Support Rating Scale (SSRS) (21), which includes 10 items measuring three dimensions: subjective support (four items), objective support (three items), and support-seeking behavior (three items). (Sample item: ‘In the past, when you encountered difficulties, from what source did you receive comfort and caring?') Item scores of SSRS were simply added, generating a total support score of 12 to 66, with higher scores indicating greater social support. This scale is widely used and has good reliability and validity in Chinese samples (22, 23). In this study, Cronbach's alpha of the scale was 0.80.

#### Resilience

Resilience was assessed using the Chinese version of the Connor–Davidson Resilience Scale (CD-RISC) (24), which includes 25 items measuring three dimensions: tenacity (13 items), strength (eight items), and optimism (four items). (Sample item: ‘When things look hopeless, I don't give up'). Each item was rated on a 5-point Likert scale ranging from 1 (never) to 4 (always). The total scale score ranges from 0 to 100, with a higher score indicating greater resilience. The 25-item CD-RISC has been shown to have good psychometric properties in Chinese people (24). In the current study, Cronbach's alpha of the scale was 0.93.

#### Postnatal depression

PPD was assessed using the Edinburgh Postnatal Depression Scale (EPDS) (25), which includes 10 items to measure self-reported symptoms associated with depression experienced in the past week. (Sample item: ‘I have felt scared or panicky for no good reason'). Items are rated on a 4-point Likert scale, ranging from 0 to 30, with higher scores indicating increased depressive symptoms. Scores of at least 13 indicate a likelihood of suffering from depression. In the present study, Cronbach's α for the scale was 0.82.

#### Parenting sense of competence

PSOC was assessed using the PSOC scale (26). The 17 items of this scale comprise two subscales, with eight items in the efficacy subscale and nine items in the satisfaction subscale. (Sample item: ‘How satisfied are you with your ability to care for and protect your child?'). Each item is rated on a 6-point Likert scale ranging from 1 (strongly disagree) to 6 (strongly agree). The total scale score ranges from 17 to 102, with higher the scores indicating a higher the sense of competence and satisfaction in parenting. In the present study, Cronbach's α for the scale was 0.73.

Demographic (e.g., age, education level) and clinical (e.g., parity, mode of birth) characteristics of puerperal women were collected using a self-designed questionnaire.

### Statistical analysis

Descriptive statistics and Pearson correlation analysis were conducted using IBM SPSS Statistics 26.0. The SPSS Macro PROCESS developed by Hayes (27) was used to test the mediating effects. Model 4 in the PROCESS template was used to examine the multiple mediating effects of resilience and PPD. In addition, we used the bootstrapping method randomly sampled 5,000 times with 95% confidence intervals (CIs) to test the significance of indirect effects. If CIs did not include zero, then the effect was regarded as significant.

## Results

### Sample characteristics

A total of 248 pregnant women participated in the study, and 234 valid questionnaires were returned, with an effective recovery rate of 94.35%. Table 1 shows the characteristics of the participants.

**Table 1:** Demographic information.

**Characteristics**	**Pregnant women (*N* = 234)**
	***$\bar{x}\pm$s* / *n* (%)**
**Age (years)**	29.21 ± 3.91
**Education level**	
≤ High school	51 (21.8%)
≥Junior college	183 (78.2%)
**Parity**	
Primiparous	150 (64.1%)
Maternity	84 (35.9%)
**Mode of birth**	
Vaginal delivery	160 (68.4%)
Cesarean section	74 (31.6%)

### Preliminary analyses

Descriptive statistics and correlations are shown in Table 2. The results showed that social support is positively correlated with PSOC (*r* = 0.318, *p* < 0.01). Resilience is positively correlated with both social support and PSOC (all *p* < 0.01). PPD is negatively correlated with both social support and PSOC (all *p* < 0.01). Resilience is negatively correlated with PPD (*r* = −0.409, *p* < 0.01). To test for common method bias, we conducted Harman's single-factor test, which is one of the most widely used techniques (28). If a single factor accounts for over 50% of the variance, then common method variance is likely to be present. In this case, the results indicated that only 20.73% of the variance was explained by one factor, indicating that results are not substantially influenced by common method bias.

**Table 2:** Correlation for all variables (N = 234).

	**Mean**	**SD**	**SSRS**	**CD-RISC**	**EPDS**	**PSOC**
SSRS	41.48	6.77	1			
CD-RISC	69.55	14.39	0.380**	1		
EPDS	8.27	4.38	−0.255**	−0.409**	1	
PSOC	67.79	7.72	0.318**	0.370**	−0.395**	1

SSRS, Social Support Rating Scale; CD-RISC, Chinese version of Connor–Davidson Resilience Scale; EPDS, Edinburgh Postnatal Depression Scale; PSOC, Parenting Sense of Competence Scale. ^**^ p < 0.01.

### Multiple mediating analysis

**Figure 2:**
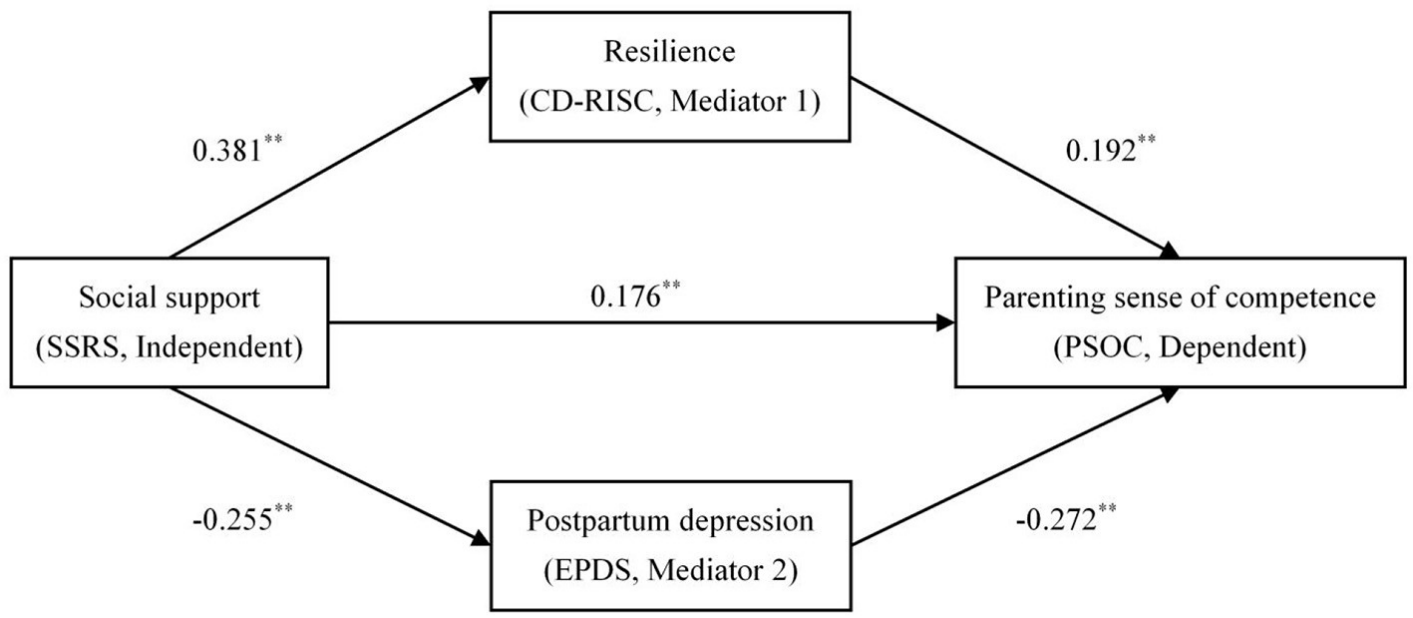
Multiple mediating paths between social support and parenting sense of competence. **p < 0.01.

After testing the correlations, a multiple mediating analysis in the relationship between social support and PSOC was conducted (Table 3 and Figure 2). The results showed that social support is positively correlated with PSOC and resilience (all *p* < 0.01), but negatively correlated with PPD (β = −0.255, *p* < 0.01).

**Table 3:** Multiple mediating models between social support and parenting sense of competence.

**Predictors**	**Model 1 (PSOC)**		**Model 2 (CD-RISC)**		**Model 3 (EPDS)**		**Model 4 (PSOC)**	
	**β**	***t***	**β**	***t***	***B***	***t***	**β**	***t***
SSRS	0.318	5.113**	0.381	6.267**	−0.255	−4.015**	0.176	2.802**
CD-RISC							0.192	2.881**
EPDS							−0.272	−4.266**
*R*	0.318	0.38	0.255	0.484
*R^2^*	0.101	0.145	0.065	0.234
*F*	26.146**	39.271**	16.124**	23.459**

^**^ p < 0.01.

With the addition of resilience and PPD for PSOC, the regression coefficient of social support is reduced to 0.176 (*p* < 0.01). Moreover, resilience is positively correlated with PSOC (β = 0.192, *p* < 0.01), and PPD is negatively correlated with PSOC (β = −0.272, *p* < 0.01). Thus, this study found that resilience and PPD mediate the relationship between social support and PSOC.

Specifically, the bootstrap method indicated that the mediation effect of resilience (effect = 0.073, *SE* = 0.031, 95% CI = [0.014, 0.136]) and PPD (effect = 0.069, *SE* = 0.024, 95% CI = [0.029, 0.121]), and the total mediation effect are all significant (effect = 0.142, *SE* = 0.038, 95% CI = [0.072, 0.220]). Moreover, these factors separately accounted for 22.96%, 21.70% and 44.65% of the total effect, respectively (Table 4).

**Table 4:** Multiple mediating paths between social support and parenting sense of competence.

	**Effect**	**BootSE**	**95% CI**		**Relative effect**
			**Lower**	**Upper**	
Direct effect	0.176	0.063	0.052	0.300	55.35%
Total indirect effect	0.142	0.038	0.072	0.220	44.65%
Indirect effect 1	0.073	0.031	0.014	0.136	22.96%
Indirect effect 2	0.069	0.024	0.029	0.121	21.70%
Indirect effect 1 – Indirect effect 2	0.004	0.04	−0.077	0.079	

Indirect effect 1 = SSRS → CD-RISC → PSOC; Indirect effect 2 = SSRS → EPDS → PSOC; Indirect effect 1 – Indirect effect 2 = Pairwise contrasts between indirect effects.

Table 4 shows that the pairwise contrast between the two indirect effects is not significant (effect = 0.004, *SE* = 0.04, 95% CI = [−0.077, 0.079]). The difference between the two pathways suggests that maternal resilience and PPD play different roles in the relationship between social support and PSOC.

## Discussion

This study found that puerperal women had a moderate level of PSOC, which was lower than that previously reported for mothers from both Guangzhou city (8) and Jiaxing city (29). Further analysis found that the parenting satisfaction subscale score in our study was comparable with that in these earlier studies, while the parenting efficacy subscale score was lower. We found that most of the puerperal women in our study had parents or parents-in-law to assist in taking care of the newborn, suggesting that the mothers were highly dependent on their original families. This may lead to the loss of a mother's growth opportunity to switch to a caregiver role, and affect the parenting efficacy of puerperal women (30). On the other hand, Guangzhou and Jiaxing are cities with a relatively dense population of non-local residents (29, 31), suggesting that direct maternal participation in childcare may be higher than in Yangzhou. This may explain why the scores for parenting efficacy and PSOC are higher in these cities, although further studies are needed for confirmation. Our results also suggest that puerperal women are not well fulfilled in their maternal role. Many mothers experienced feelings of inadequacy associated with their lack of knowledge of infant behavior and infant care. Obstetric nurses should therefore focus on the psychology and behavior of puerpera, and assess the difficulties during child-rearing in a timely manner. Patient communication and education are necessary to promote maternal competence in the puerperium.

This study confirms the direct effect of social support on PSOC. The results showed that puerperal women who received more social support present higher maternal role competence, which is consistent with a study from the US (32). According to Bandura, social persuasion and observation of the parenting behavior of significant support figures are important sources of information affecting maternal role competence (33). Social support is like a barrier that protects people against the potential effects of stressful life events and enables them to cope with difficulties (10). Social support increases parental role competence by providing encouragement and resources for childcare (34). A supportive couple relationship can help mothers adopt a more positive parenting style, making them more motivated to deal with the tasks and challenges during the parenting process, reducing parenting pressure, and improving parenting competence (35). Moreover, young mothers who are able to receive support from their mothers have a higher PSOC, while greater family support enables mothers to have more positive parenting experiences (36). Culturally competent healthcare intervention should be developed during early motherhood to promote perceived social support and parenting self-efficacy for new mothers (9). Additionally, knowledge of infant care has been found to play a significant role in building women's sense of maternal competency, particularly for first-time mothers (37). Typically, new mothers engage in web-based forums to seek help and support during the postpartum period, and web-based communities play an important role in maternal peer social support, information seeking, and early parenting practices (38).

Our study confirms the mediating role of PPD in social support and PSOC among puerperal women. Taking care of the newborn in the early postpartum period is challenging for the mother owing to pain, sleep deprivation, and hormonal imbalance (39). Thus, social support is greatly needed from family, friends, and others. Without sufficient social support, women may be at high risk of PPD. High risk of PPD is also associated with low levels of PSOC, as was also shown in a previous study by Martinez-Torteya (6). Postpartum depressive symptoms may drive mothers to avoid interactions with their infants and increase their perceived stress and challenges in the process of parenting, resulting in increased parenting stress (35). The increase in parenting stress has a relatively negative impact on mothers' beliefs of their maternal role competence (35). Thus, a suggestion is to educate families on the importance of social support and improve this in every aspect of health care in order to prevent PPD and enhance PSOC.

Our results support previous findings that resilience acts as a mediator between social support and PSOC among puerperal women. The Kumpfer resilience framework indicates that individuals' adaptation to the external environment is affected by the external environment and interaction between individuals and the environment (40). The higher the level of social support; the higher the level of resilience among individuals. Social support can provide individuals with information, emotional and material help, and can help individuals facing difficulties and setbacks, thereby improving resilience (41). Women with higher levels of resilience can mobilize resources around them to solve problems actively when faced with stress or unexpected life events, and adjust to the negative effects of stress with stronger adaptability, helping achieve the maternal role. This finding aligns with the results of Martinez-Torteya who reported that high resilience is associated with high levels of PSOC (6). Conversely, individuals with low resilience are easily affected by stress, which is accompanied by higher levels of anxiety, depression, and maladaptation (42), thereby affecting their parenting efficacy and parenting satisfaction. The results of this study also suggest that medical staff should focus on the assessment of maternal resilience. Additionally, we can use peer support, stress management, and positive psychological interventions to intervene as soon as possible in cases of pregnant women with low resilience to improve their parenting competence.

This study has some limitations. First, puerperal women were included by convenience sampling in just one obstetrics clinic. Hence, the results of this study should be interpreted with caution. Second, as most participants were women with high levels of education, the findings may not be generalizable to those who have limited education or live in rural areas. Third, no investigation was undertaken in this study into whether or not the pregnancy was wanted; this should be investigated and analyzed separately to verify the findings.

## Conclusion

Our study provides information on the mechanisms underlying psychosocial factors and maternal role competence. The model confirms the mediating role of resilience and PPD in the relationship between social support and PSOC in puerperal women.

This study also suggests that medical staff should consider mobilizing a maternal social support system and focus on the psychological health of puerperal women. Exploring the potential advantages of puerperal women in improving resilience and preventing PPD are potentially effective methods that may be used to improve mothers' parenting competence.

## Data availability statement

The raw data supporting the conclusions of this article will be made available by the authors, without undue reservation.

## Ethics statement

The studies involving human participants were reviewed and approved by School of Nursing, Yangzhou University (Ethics Number: YZUHL2020009). The patients/participants provided their written informed consent to participate in this study.

## Author contributions

XS conceived, planned, and designed the study. XS and LL collected the data. CN and YL supervised the project. XS and SG wrote the first draft of the manuscript. All authors contributed to the article and approved the submitted version.

## Funding

This study was supported by grants from the ‘Huxin Fund' project of Jiangsu Key Laboratory of Zoonotic Diseases (HX20015), Key R&D Projects of Yangzhou (YZ2021067), and Guangzhou Science and Technology Plan Project (202206060004).

## Conflict of interest

The authors declare that the research was conducted in the absence of any commercial or financial relationships that could be construed as a potential conflict of interest.

## Publisher's note

All claims expressed in this article are solely those of the authors and do not necessarily represent those of their affiliated organizations, or those of the publisher, the editors and the reviewers. Any product that may be evaluated in this article, or claim that may be made by its manufacturer, is not guaranteed or endorsed by the publisher.
